# A Novel Deep Learning Model as a Donor–Recipient Matching Tool to Predict Survival after Liver Transplantation

**DOI:** 10.3390/jcm11216422

**Published:** 2022-10-29

**Authors:** Nikolaus Börner, Markus B. Schoenberg, Philipp Pöschke, Christian Heiliger, Sven Jacob, Dominik Koch, Benedikt Pöllmann, Moritz Drefs, Dionysios Koliogiannis, Christian Böhm, Konrad W. Karcz, Jens Werner, Markus Guba

**Affiliations:** 1 Department of General, Visceral and Transplantation Surgery, Ludwig-Maximilians-University, 81377 Munich, Germany; 2 Institute of Informatics, Ludwig-Maximilians-University, 81377 Munich, Germany

**Keywords:** liver transplantation, machine learning, quality management, data management

## Abstract

Background: The “digital era” in the field of medicine is the new “here and now”. Artificial intelligence has entered many fields of medicine and is recently emerging in the field of organ transplantation. Solid organs remain a scarce resource. Being able to predict the outcome after liver transplantation promises to solve one of the long-standing problems within organ transplantation. What is the perfect donor recipient match? Within this work we developed and validated a novel deep-learning-based donor–recipient allocation system for liver transplantation. Method: In this study we used data collected from all liver transplant patients between 2004 and 2019 at the university transplantation centre in Munich. We aimed to design a transparent and interpretable deep learning framework to predict the outcome after liver transplantation. An individually designed neural network was developed to meet the unique requirements of transplantation data. The metrics used to determine the model quality and its level of performance are accuracy, cross-entropy loss, and F1 score as well as AUC score. Results: A total of 529 transplantations with a total of 1058 matching donor and recipient observations were added into the database. The combined prediction of all outcome parameters was 95.8% accurate (cross-entropy loss of 0.042). The prediction of death within the hospital was 94.3% accurate (cross-entropy loss of 0.057). The overall F1 score was 0.899 on average, whereas the overall AUC score was 0.940. Conclusion: With the achieved results, the network serves as a reliable tool to predict survival. It adds new insight into the potential of deep learning to assist medical decisions. Especially in the field of transplantation, an AUC Score of 94% is very valuable. This neuronal network is unique as it utilizes transparent and easily interpretable data to predict the outcome after liver transplantation. Further validation must be performed prior to utilization in a clinical context.

## 1. Introduction

Liver-related death accounts for approximately 2 million deaths per year and is continuously increasing [1]. Combined, liver cirrhosis and liver cancer account for 3.5% of all death worldwide [2]. Liver transplantation (LT) can be a curative and life prolonging therapy for patients with end-stage liver disease. Furthermore, the post-transplant survival of the patient and the survival and functionality of the graft is continuously increasing [3]. However, as we live in a time of organ shortage there is a large gap between supply and demand [4]. Contrary to kidney transplantation, the guiding principle for the allocation of livers is urgency. This urgency is estimated by the model for end-stage liver disease (MELD) [5]. Aside from being an imperfect estimation of the severity of the underlying diseases, this urgency-based system can lead to transplantations in increasingly futile cases and is in danger of manipulation [6,7]. There are many ordinal and deontological allocation concepts. However, none of these, at first sometimes logical concepts (lottery, first-come-first-served, etc.), can fulfil all requirements (equal treatment, maximizing benefit for all, maximizing the benefit for the individual patient and respecting autonomy) for a truly fair allocation concept. In a recent publication we showed that a utility-based system that has the goal of maximizing gain-of-survival after transplantation could potentially eliminate futile transplantations [7]. For this, however, almost perfect prediction of survival is needed. In recent years, multiple simple models such as the donor risk index (DRI) [8], survival outcome following liver transplantation (SOFT) [9], balance of risk (BAR) [10] or the donor age x recipient MELD (D -MELD) [11] have been developed. However, none appear to be vastly superior in predicting outcome after liver transplantation [12]. It seems that artificial intelligence (AI) might be suited for that [13]. AI has shown promising results in multiple medical fields [14,15,16]. It is also a competent method of choice for reducing human innate subjectivity [17]. Recently, AI has also been increasingly applied to data from transplantations since predicting outcome based on donor and recipient data is especially hard [18,19]. Results from these studies are promising. A recent review underlined, that artificial neural networks are the most common algorithms to be used in transplantation data sets. The authors point out that neural networks are especially suited since they are more flexible than older score-based systems. Also, they conclude that more accurate neural networks could aid in in better allocation by taking more variables into account [20].

In this study, we report on the development and testing of a novel deep learning model for the prediction of overall survival at different timepoints after transplantation.

## 2. Materials and Methods

### 2.1. Data Selection and Study Population

In this single-centre study, we used data collected from all liver transplant patients between 2004 and 2019 at the university transplant centre in Munich/Erlangen. Ethical approval was obtained from the institutional review board (EK 19–395) at the Ludwig-Maximilian University in Munich. The need for an informed consent was waived by the institutional review board. Patients were selected by having both the donor and recipient values present. Data from a total of 1058 individuals were included. To present a transparent and interpretable model we included variables that are globally available and belong to the “standard” panel of recorded data. Recipient data consisted of demographic data (age, sex, gender, BMI, etc.), the underlying disease (Cirrhosis, etc.), disease features (Ascites, etc.), the MELD score and fourteen laboratory values. Donor data consisted of demographic data (age, sex, etc.), living or deceased donor, cause of death, reanimation of the deceased, donor risk index and fourteen laboratory values. We selected donor variables that were available prior to organ allocation since this reflects the clinical reality. Any donor variables that were only available after Tx were neglected. Importantly, we also included transplant-specific data that is not directly related to donor or recipient. Transplant-specific data consisted of ischemia time, full or split donation, distance organ travelled to transplantation location and graft quality. The detailed recipient, donor and transplant-specific datapoints are outlined in Table 1, Table 2 and Table 3.

### 2.2. Missing Data

Transplant data is generally heterogeneous and therefore challenges traditional statistical models. Since the organ is donated by a person who is largely independent from the recipient, the missing data needs to be calculated independently from one another. Further location data and laboratory markers may require different algorithms to estimate the most accurate value missing. Therefore, we developed and validated a novel multidimensional medical combined imputation (MMCI) algorithm to analyse this multifaceted and segmented dataset. The MMCI is a pipeline of interconnected imputation methods to impute segmented data with the highest accuracy. We have tested and validated the imputation mechanism on two different complete datasets. For both datasets, the most established imputation methods were tested, and accuracy (ACC) was compared with the novel MMCI. The model outperformed well-established imputation mechanisms such as missforrest, k-NN and MICE.

### 2.3. Model Development

An individually designed neural network was developed to meet the requirements of the data. A neural network, in general, is an arrangement of linear and non-linear modules that enable a network of nodes to intercommunicate and learn using the training data. The training data includes input data (variables) and the outcome which is to be predicted. To understand the correlation and causality within the data, the network needs several layers between input and outcome. At every node (decision point) data importance is weighted. An important hyperparameter to tune is defining the correct size of the layers and their depth. A lack of layers and nodes leads to the network underrepresenting individual data points and thus an underfitting occurs. Underfitting describes a situation in which the model is too rigid to be able to predict the outcome. However, when the model is oversized then it could lead to overfitting. Overfitting describes the situation when the model is trained so specifically that it is only able to predict the dataset it was trained on and is therefore not generalizable. To avoid this, four hyperparameters were introduced to monitor the development of the learning progress and to adjust the network in an iterative adaptation process. The goal was to scale the network to find the right balance between over- and underfitting. To measure this, the metrics accuracy, cross-entropy loss, F1 Score and AUC Score were utilized for monitoring. Accuracy reflects how often the network was correct in its prediction. Cross-entropy loss describes how far the prediction and the true value are divergent. F1 score is a weighted mean of the precision and recall metrics. It is especially helpful when a classifier “X” has a high precision and another classifier “Y” has a high recall. In this scenario, the F1 score helps to compare the average results of both models within one metric. Lastly, the AUC tells us how well the model can distinguish between different classes. (Detailed definitions are in Supplementary S1.b-Metric Definitions).

**Figure 1:**
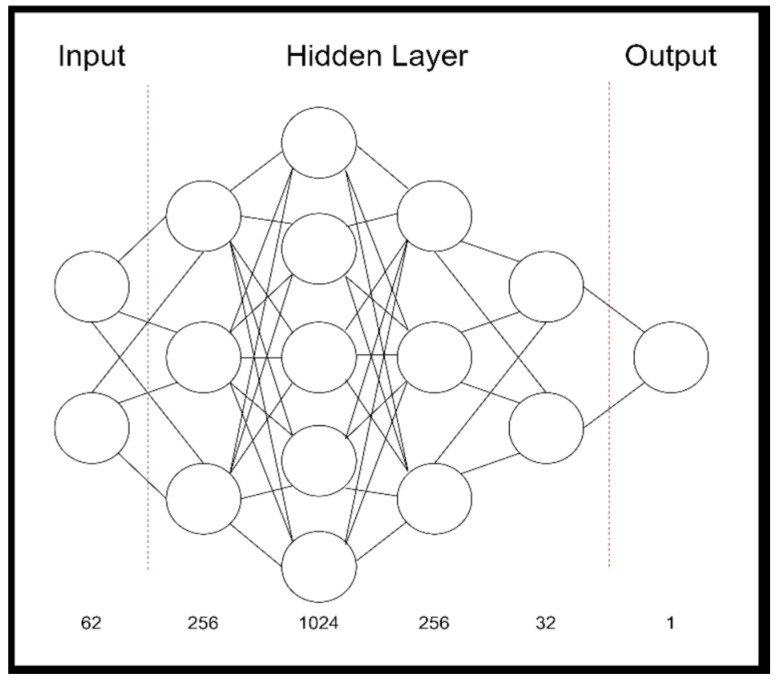
Simple schematic display of the deep neuronal network utilized.

After imputation and before training and cross-validation of the algorithm, the study cohort was split 8:1:1. This split ratio describes the training data (80%), the cross-validation data (10%) and finally the separate test data (10%). We used this common split ratio to allow the algorithm to train on as much data as possible to combat the phenomena of over- and underfitting. With a total of 529 transplantations in the study group, the training dataset (including 80% training data and 10% cross-validation data) included n = 477 and the test dataset n = 52 transplantations. After separation, the test dataset remained untouched throughout the analysis and was only used for testing the final model. With the given amount of 62 variables and 478 transplantations (956 observations from donors and recipients) inside the training dataset, the depth of the network was defined in six layers. A simple schematic display of the model is outlined in Figure 1.

### 2.4. Code Description

It is necessary to understand that the whole framework of this code has been built to serve as a dynamic usable structure for all types of transplant data. Therefore, it is built in a modular way where the user can train this specialized neural network on any transplant. The basic description is outlined in Supplementary S1.a. “Basic-Code”.

### 2.5. Outcome Parameter

Accuracy was defined as the primary metric for training and testing of the prediction for the survival rate. The last layer of the network is the outcome. The network learns and evaluates itself during the training phase. Due to the fact that different timepoints of survival were evaluated, a subdivision of the data within the algorithm was made to increase the precision of the prediction. This subdivision related to death within 48 h, in-hospital mortality, 3-month, 6-month, 9-month and 12-month survival rates and death within the follow-up period.

To gain value from this network, the allocation needs to be conducted with one current donor for all possible recipients that are available. Based on having a list of possible recipients who are waiting for an organ and the new availability of one donor in the process of organ-extraction, a real-time allocation needs to take place to determine what the best mapping is. Therefore, out of the evaluation dataset, one patient is extracted and mapped onto the whole recipient dataset. Thus, a prediction is made based on the previous attributes that were necessary for determining the rate of survival.

## 3. Results

### 3.1. Demographic Data Characteristics

A total of 529 transplantations were included from 2004 to 2019. For these, 529 matching donor and recipient observations were added into the database. The demographic and clinical data for the transplanted patients are listed in Table 1. Accepted organs were 321.56 ± 210.99 km distant from the university transplantation centre in Munich. Consequently, the cold ischemia time is relatively high at 630.69 ± 156.61 min (Table 2). Donors were 54.79 ± 16.27 years old. Overall, they had a calculated donor risk index of 1.98 ± 0.43. Albumin levels were 27.86 ± 6.46 g/L. Notably, when comparing the recipient data, inflammation parameters were increased with leukocytes at 13.85 ± 5.95 G/L and CRP 14.78 ± 10.72 mg/dL. All data are listed in Table 3.

Variables were compared between the training and the test datasets. Regarding recipients, all demographic disease-specific variables showed no significant difference. In the comparison of the laboratory values, only potassium levels were shown to be significantly different between the datasets.

In the comparison of the donor data. The DRI was higher in the training dataset (*p* = 0.0095).

### 3.2. Algorithm Performance

The metrics used to determine the model quality and its level of performance are accuracy, cross-entropy loss, F1 score and AUC score. By splitting the initial data set with a ratio of 8:1:1, where the first nine parts were used to train the network, the evaluation can take place on completely unseen data that were initially randomly sorted out. The distribution of outcome data between the training set and evaluation set is represented in Supplementary S1.c Figures S1 and S2.

The overall results given by the metrics calculated through an average of all outcome parameters show 0.958 (95.8%) accuracy with a cross-entropy loss of 0.042. The combined F1 score was 0.899, whereas the AUC score was 0.940. For better visibility, the summarized results are shown in Table 4.

## 4. Discussion

This study represents a novel deep-learning-based prediction model for survival after liver transplantation. The model was trained on 529 transplantations including 1048 donors and recipients. Further, it aims to be interpretable and transparent, especially in its process of data utilization. It managed to perform with an AUC of 0.940 that, in a clinical context, represents a very strong prediction. We chose this method because of the nature of the dynamic interaction between the donor and recipient. It is built in a modular way, where the user can train this specialized neural network on any transplant data with little effort. Previous studies have applied similar methods to accomplish predictions of outcome. Since we did not utilize the same data and used different outcome parameters a direct comparison between different prediction models is difficult. However, Ayllon et al. achieved an AUC of 0.82 for prediction of 12-month survival after liver transplantation [20]. Ershoff et al. achieved an AUC of 0.703 [21] for the prediction of 90-day post-transplant mortality. Our model achieved comparable and higher accuracies to those shown above. Even though these models also seem to be performing reasonably well, they can only predict one timepoint, whereas the model presented here can predict multiple timepoints.

The European General Data Protection Regulation of 2018 stated reasonable concerns with black-box predictions. The concerns not only include the opaqueness of the model itself but also the necessity to have control over the data, the processing and the interpretation of the results obtained [22]. Our data selection and processing were specifically set out to meet these concerns. As missing data is omnipresent within medical archives, we developed our own imputation method, which proved to be more accurate than readily known imputation algorithms (Boerner et al. under review).

Regarding the interpretation of the resulting predictions, we propose to create an AI-assisted utility-based allocation concept. AI offers the chance of utilizing the vast amount of data in the field of transplantation to optimize organ utility. The state-of-the-art allocation target metrics such as the DRI, MELD score, SOFT score, and BAR score offer some success in predicting the most favourable outcome. However, these scores are criticized for being inaccurate, untransparent and static [23,24,25]. An AI-assisted utility-based allocation concept using gain-of-survival as the target metric would be more flexible and could represent the best approximation towards a perfect allocation practice [7,26].

This study has some limitations that are inherent to its design. First of all, this is a retrospective study with data from two German transplant centres. We have mitigated the possible biases by utilizing cross-validation to make our results more generalizable. Multiple models have been developed over the past years and have aided the process of discussion on how to incorporate machine learning and neuronal networks into our clinical decision process [27,28]. As mentioned above, we have strived for maximum transparency, however, the very nature of a black-box model could not be fundamentally changed.

This study is intended as a proof of concept. It represents a novel deep learning model that was trained and tested. Such a model could potentially be used as a part of a utility-based allocation concept. Before this model or any of its kind can be used as a bedside tool, the results need to be externally confirmed in a randomized clinical trial, ideally in a multicentre setting.

## Figures and Tables

**Table 1:** Study data from the recipient study cohort before transplantation. Training and test data are compared.

Characteristics	Study Cohort	Training Data	Test Data	Training vs. Test
	*n* = 529	*n* = 477	*n* = 52	*p*-Value
**Demographics**				
Age at operation in years, mean ± SD	50.28 ± 12.29	50.06 ± 12.46	52.31 ± 10.58	0.2113
Male/Female	357/172	318/159	39/13	0.2755
Height (m), mean ± SD	1.73 ± 0.10	1.73 ± 0.10	1.73 ± 0.09	0.9754
Weight (kg), mean ± SD	77.57 ± 16.39	77.79 ± 16.36	75.58 ± 16.66	0.3543
BMI, mean ± SD	25.67 ± 4.59	25.74 ± 4.57	25.03 ± 4.44	0.2903
**Liver Disease features**				
Ascites, Y/N	332/197	301/176	31/21	0.6518
Encephalopathy, Y/N	216/313	194/283	22/30	0.8822
Dialysis, Y/N	77/452	72/407	5/47	0.2921
MELD, mean ± SD	23.79 ± 11.08	23.86 ± 11.16	23.17 ± 10.50	0.6710
Allocation MELD, mean ± SD	27.75 ± 8.55	27.83± 8.66	27.15 ± 7.64	0.5912
**Laboratory Values**				
Na mmol/L, mean ± SD	135.98 ± 5.42	135.98 ± 5.43	135.98 ± 5.38	0.9983
K mmol/L, mean ± SD	4.10 ± 0.50	4.11 ± 0.49	3.95 ± 0.55	0.0268
Bilirubin mg/dL, mean ± SD	12.12 ± 13.56	12.02 ± 13.32	12.97 ± 15.83	0.6296
Albumin g/L, mean ± SD	3.15 ± 0.67	3.15 ± 0.68	3.16 ± 0.60	0.8627
ALT U/L, mean ± SD	328.94 ± 876.02	306 ± 829.33	421.81 ± 1023.03	0.0967
AST U/L, mean ± SD	454.85 ± 1318.16	389.63 ± 1125.18	684.92 ± 1854.65	0.3536
GGT U/L, mean ± SD	141.45 ± 186.77	140.23 ± 186.29	144.37 ± 189.98	0.8796
AP U/L, mean ± SD	231.38 ± 252.37	225.67 ± 251.54	246.48 ± 237.75	0.5693
Haemoglobin g/dL, mean ± SD	10.58 ± 2.50	10.60 ± 2.50	10.43 ± 2.47	0.6348
INR, mean ± SD	1.76 ± 0.90	1.77 ± 0.94	1.62 ± 0.51	0.2541
Creatinine mg/dL, mean ± SD	1.66 ± 1.16	1.65 ± 1.14	1.83 ± 1.30	0.2843
CRP mg/dL, mean ± SD	2.51 ± 3.58	2.50 ± 3.64	2.60 ± 3.09	0.8481
Leukocytes 10^6^/L, mean ± SD	8.15 ± 6.47	8.22 ± 6.66	7.50 ± 4.37	0.4426
Platelets 10^6^/L, mean ± SD	100.27 ± 74.17	100.49 ± 75.54	98.17 ± 60.68	0.8305

**Table 2:** Study data regarding variables during transplantation.

Characteristic	Study Cohort	Training Data	Test Data	Training vs. Test
	*n* = 529	*n* = 477	*n* = 52	*p*-Value
Cold ischemia time (min) ± SD	630.69 ± 156.61	634.28 ± 159.66	597.77 ± 121.49	0.1104
Full/split liver ± SD	499/30	447/30	52/0	0.0607
Distance explanation to transplantation (km) ± SD	312.56 ± 210.99	328.52 ± 210.31	257.73 ± 208.38	0.0215
Duration of stay (days) ± SD	45.15 ± 39.87	44.79 ± 39.64	48.42 ± 42.13	0.5334

**Table 3:** Study data from the donor study cohort before transplantation. Training and test data are compared.

Characteristics	Study Cohort	Training Data	Test Data	Training vs. Test
	*n* = 529	*n* = 477	*n* = 52	*p*-Value
**Demographics**				
Age at operation in years, mean ± SD	54.79 ± 16.27	54.68 ± 16.21	55.71 ± 16.87	0.6669
Male/Female	271/258	241/236	30/22	0.3814
Height (m), mean ± SD	1.72 ± 0.09	1.72 ± 0.09	1.73 ± 0.08	0.6766
Weight (kg), mean ± SD	77.81 ± 14.71	77.88 ± 14.94	77.21 ± 12.48	0.7545
Donor reanimation	136 (25.71%)	122 (25.58%)	14 (26.92%)	0.2429
Donor risk index	1.98 ± 0.43	1.98 ± 0.44	1.82 ± 0.37	0.0095
**Laboratory Values**				
Na mmol/L, mean ± SD	147.9 ± 8.18	147.93 ± 8.10	147.60 ± 8.93	0.7803
K mmol/L, mean ± SD	4.2 ± 0.56	4.21 ± 0.57	4.10 ± 0.50	0.1627
Bilirubin mg/dL, mean ± SD	0.69 ± 0.4	0.69 ± 0.4	0.66 ± 0.43	0.6129
Albumin g/L, mean ± SD	27.86 ± 6.46	27.97 ± 6.44	26.82 ± 6.61	0.2242
ALT U/L, mean ± SD	65.72 ± 132.3	65.24 ± 137.34	59.98 ± 71.44	0.7855
AST U/L, mean ± SD	83.52 ± 135.27	82.84 ± 137.30	90.46 ± 115.87	0.7002
GGT U/L, mean ± SD	83.12 ± 123.16	85.01 ± 128.15	65.86 ± 57.50	0.2874
AP U/L, mean ± SD	87.83 ± 55.3	86.92 ± 54.14	96.20 ± 64.98	0.2506
Haemoglobin g/dL, mean ± SD	10.59 ± 2.3	10.58 ± 2.31	10.72 ± 2.20	0.6897
INR, mean ± SD	1.24 ± 0.53	1.24 ± 0.54	1.24 ± 0.43	0.9953
Creatinine mg/dL, mean ± SD	1.14 ± 0.87	1.15 ± 0.88	1.12 ± 0.73	0.8153
CRP mg/dL, mean ± SD	14.78 ± 10.72	14.78 ± 10.51	14.86 ± 12.64	0.9597
Leukocytes 10^6^/L, mean ± SD	13.85 ± 5.95	13.84 ± 5.56	13.91 ± 8.79	0.9380
Platelets 10^6^/L, mean ± SD	191.02 ± 103.2	189.69 ± 103.70	203.19 ± 98.59	0.3708

**Table 4:** Results from evaluation metrics.

Metric	Death	Death within 48 h	Death in Hospital	Death within 12 Months	Results
Accuracy	99.226	94.3396	99.3396	98.113	95.745
Cross-Entropy Loss	0.0377	0.0566	0.0566	0.0189	0.0424
F1 Score	0.9412	0.842	0.842	0.9697	0.8988
AUC Score	0.9444	0.9217	0.9217	0.9706	0.9396

## Data Availability

De-identified data used in this study are not publicly available. Parties interested in the transplant data set should contact Nikolaus.boerner@med.uni-muenchen.de. The code description is outlined within the Supplementary Materials. The full IP for the mentioned novel method (MMCI) can be acquired via the co-author Philipp Pöschke (philipp.poeschke@googlemail.com).

## Supplementary Materials

The following supporting information can be downloaded at: https://www.mdpi.com/article/10.3390/jcm11216422/s1, Supplementary S1: a. Basic Code. b. Metric Definitions. c. Distribution of outcome Data Training vs. Evaluation.
