# Supplementary material for: A Novel Deep Learning Model as a Donor–Recipient Matching Tool to Predict Survival after Liver Transplantation
Source: J Clin Med. 2022 Oct 29;11(21):6422. doi: 10.3390/jcm11216422 (PMC9655123; doi:10.3390/jcm11216422)

Supplementary S1.

a. **Basic Code**
b. **Metric Definitions**
c. **Distribution of outcome Data Training vs. Evaluation**

a. “Basic Code”:

Before explaining the deep structure of the code, it is necessary to understand that the whole framework of this code has been built to serve as a dynamic usable structure for all types of transplant data. Therefore, it is built in a modular way with which the user is able to train this specialized neural network on any transplant data with only 5 lines of code after successful installation. At very first the user is pleased to enter customizable hyperparameters that are pre-defined but adaptable if needed after first iterations of training and analysing. These parameters are visualized in the visualization below.

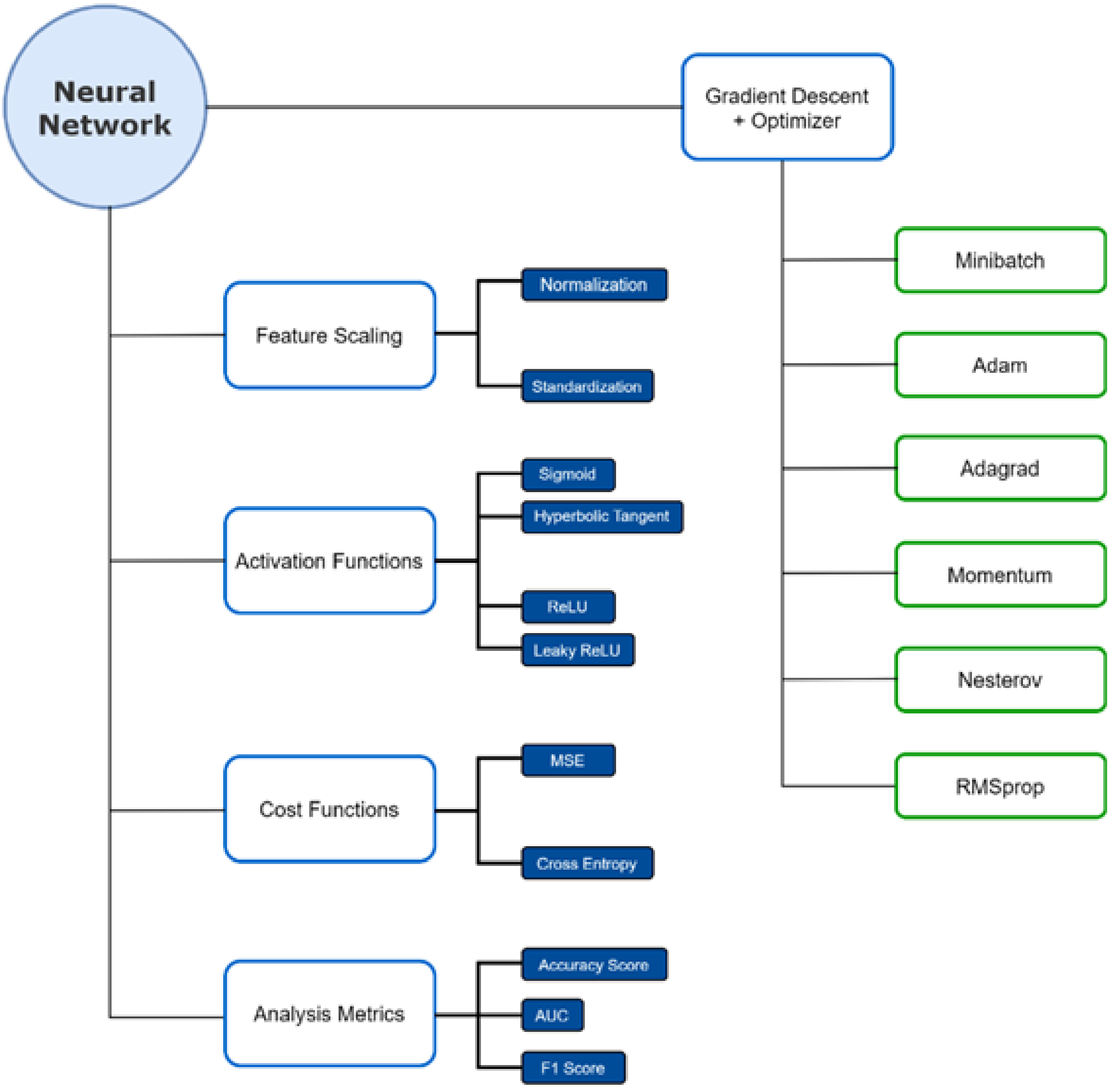

Same applies to the size of the neural network itself. It can be custom scaled in both directions – depth and length as well as the usage of the activation function per layer for all included neural network nodes.

Hyperparameter and Network Architecture

```
In [ ]:
n_eta = 3e-4            # network learning rate
n_epochs = 600001       # amount of training epochs
n_matric = 'both'       # parameter to visualize training progress regarding learning rate and epochs
n_batch = 106           # batch-size for optimizer --> related to dataset-size
n_opt = None # "adam"   # selection of optimizer for network training
n_info = True           # parameter to visualize training progress regarding optimizer and loss
n_loss = True           # selection if loss is visualized per training step
n_split = 0.1           # train-test split size
n_rand_seed = 42        # seed parameter to split training and evaluation set
n_save = True           # parameter to choose if training progress should be saved

n_architecture = [{"input_dim": 62, "output_dim": 256, "activation": "relu"},   # Input Layer
                  {"input_dim": 256, "output_dim": 1024, "activation": "relu"}, # Hidden Layer -- 1
                  {"input_dim": 1024, "output_dim": 256, "activation": "relu"}, # Hidden Layer -- 2
                  {"input_dim": 256, "output_dim": 32, "activation": "relu"},   # Hidden Layer -- 3
                  {"input_dim": 32, "output_dim": 1, "activation": "sigmoid"},  # Output Layer
                 ]
```

In the following, the real call out of the given function can take place to initiate and call the function with the given parameters. That is aiming to at first define – if set to "True" – where to store the training progress. Afterwards calling the input and output data in a split function to clearly separate the data into training and test set. According to the mathematical matrix multiplication, the subsets of data are then called "X" and "y" as a prefix. To step forward, the initialisation of the neural network can happen through calling its function. Directly in addition, the pre-defined parameters can be loaded to load the architecture in the neural network. Coming close to the end, the network can now be launched training via calling the parameters and running the training function.

Initialization and Training of the Neural Network

```
In [ ]:
n_name = "weights/neural_network_weights.json" # file-ending needs to be .json
X_train, X_test, y_train, y_test = train_test_split(X, y, test_size=n_split, random_state=n_rand_seed)

neural_network = NeuralNet() # Initialization of the neural network
neural_network.load_architecture(architecture=n_architecture, seed=n_rand_seed) # Dataset seperation into train and test
neural_network.train(X_train, y_train, X_test, y_test,
                     epochs=n_epochs, learning_rate=n_eta, minibatch_size=n_batch,
                     optimizer=n_opt, matric=n_matric, verbose=n_info,
                     show_loss=n_loss, save_weights=True, filename=n_name)
```

Finally, one can call the "analyze" function to get a detailed report regarding all final metrics as well as all network predictions to the given "X" dataset. Either to use for a detailed deep dive into the results on the training set or to use to validate evaluation data.

Printing, Analyzing & Visualizing Results

```
In [ ]:
predictions, loss , accuracy , f1 , auc = neural_network.analyze(X.T, y) # insert X and y data to analyze
```

If the neural network is trained, optimized and finally saved, its weights can be loaded each time again. In addition to all of that, with the ability of the network to replace the organ donor data in the retrospective data with a current organ donor dataset and the given list of possible organ recipients, the network can be used as well to predict the survival of the fittest regarding that given data with exactly the same above-mentioned functionality. That gives the user the ability to rank the dataset regarding the predicted survival rate (visualisation below).

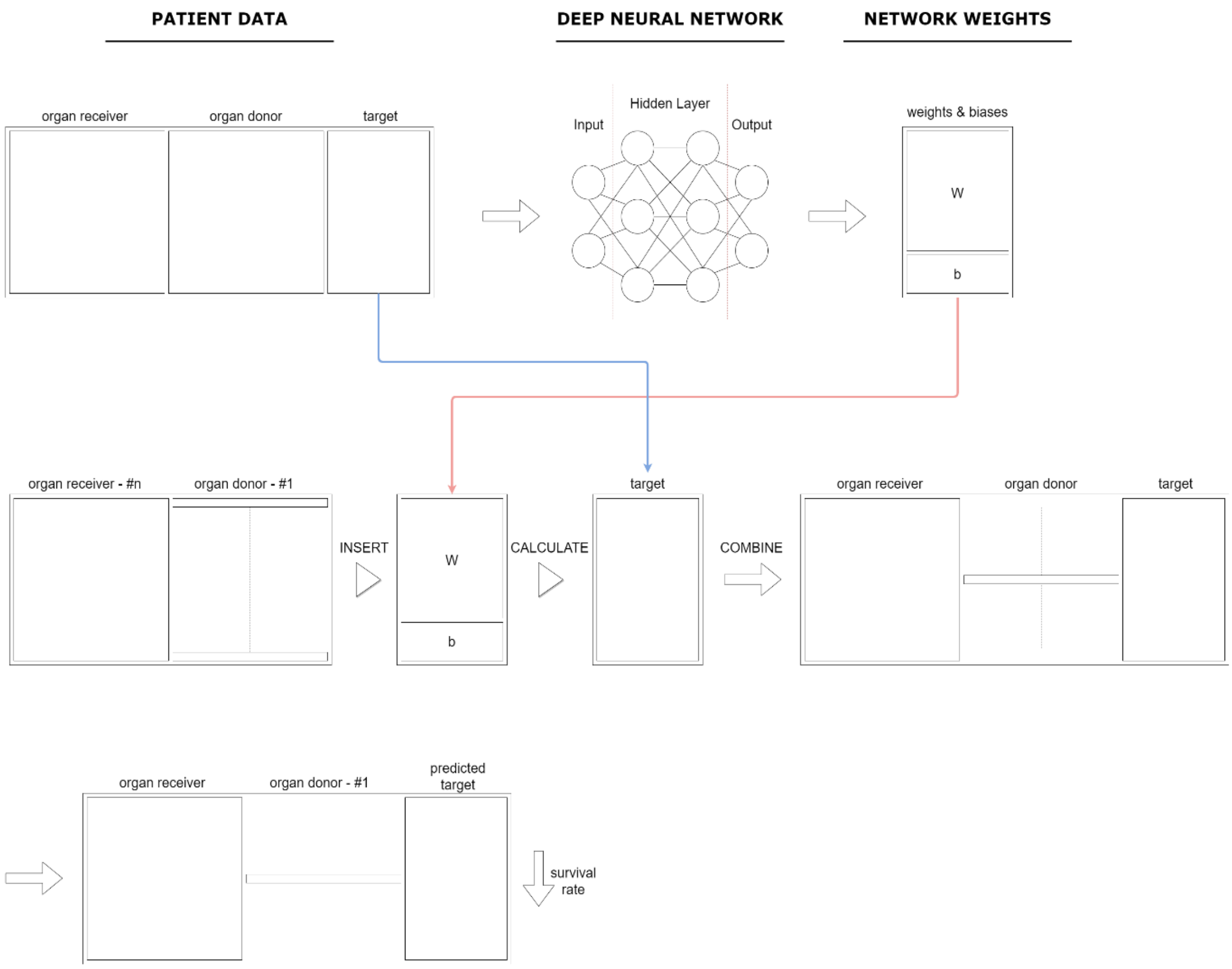

b. Metric Definitions

F-1 score:

F1 score combines precision and recall relative to a specific positive class -The F1 score can be interpreted as a weighted average of the precision and recall, where an F1 score reaches its best value at 1 and worst at 0.

$$F1 \ = 2 * \frac{precision * recall}{\text{precision} + \text{recall}}$$

Accuracy:

The accuracy is reflecting how often the network was correct and how often its was wrong. The accuracy is the calculated percentage of the difference between the predicted value and the original value.

$$Accuracy \ = 2 * \frac{true\ positive(TP) + true\ negative(TN)}{\text{true postitive} + \text{true negative} + \text{false positive(FP)} + \text{fals negative(FN)}}$$

AUC Score:

The AUC describes the area under the curve. It tells us how well the model is able to distinguish between the classes. Here 1 represents the highest reachable scale. Calculation is based on sensitivity (SE) and specificity (SP).

$$T \ = \frac{1 * \ SE}{2} = \frac{SE}{2} = \frac{TP}{2 * (\text{TP} + \text{FN})}$$

$$U = \frac{SP * \ 1}{2} = \frac{SP}{2} = \frac{TN}{2 * (\text{TN} + \text{FP})}$$

$$AUC = \text{T} + \text{U} = \frac{SE + TP}{2}$$

Cross-Entropy Loss

It measures the performance of a classification model at which the output is a value between 0 and 1. The loss is increasing if the prediction and the actual true value are diverging. Therefore, the lower the overall loss the closer the prediction is to the true value. The best outcome would be 0.

$$Loss = \frac{1}{\text{m}}(\ true * \log(pred) + (1 - true) + \log(1 - pred))$$

c. **Distribution of outcome Data Training vs. Evaluation**

Figure S1.

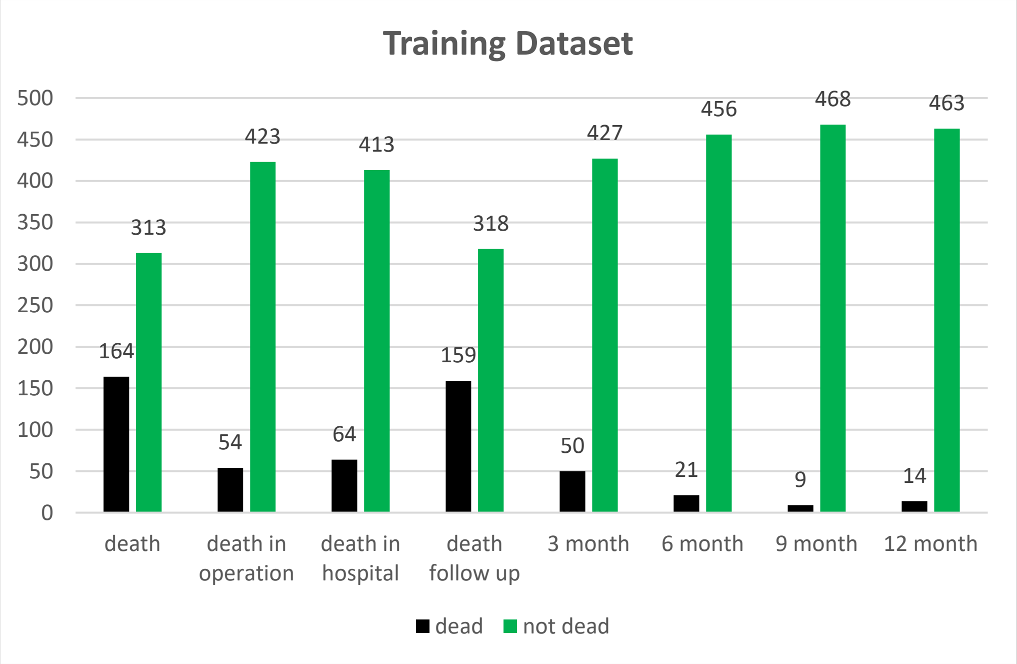

Figure S2.

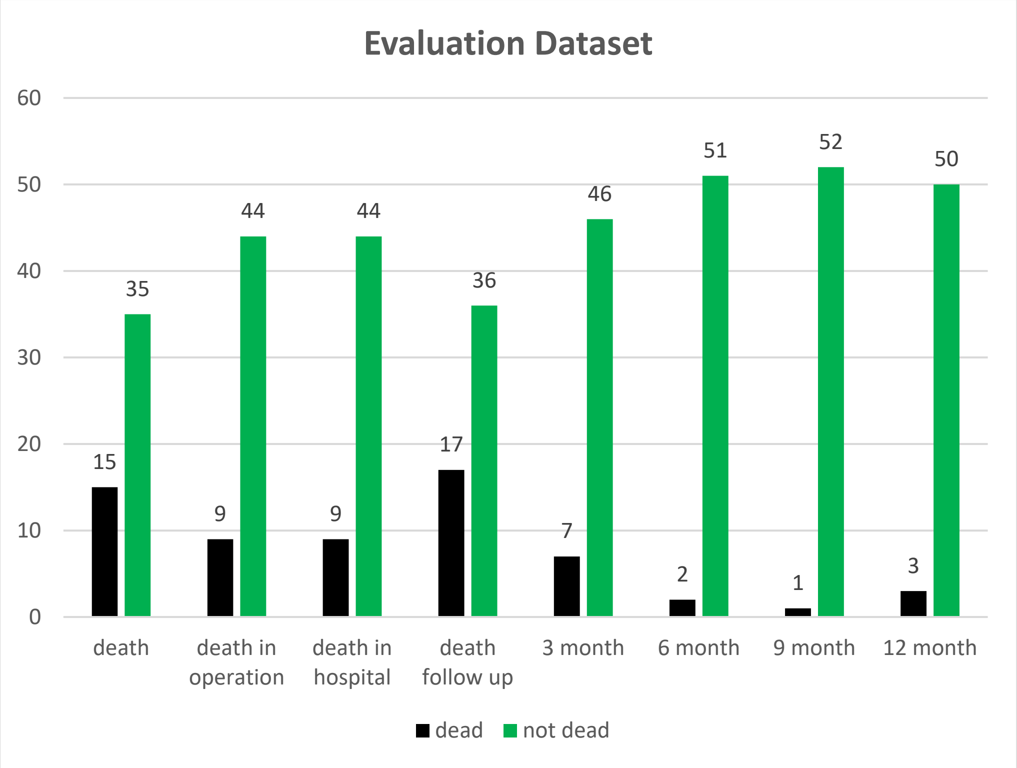

Supplement: Supplementary file 1 [file jcm-11-06422-s001.zip › jcm-1949889-supplementary.pdf]
